# “Someone to Talk to” - An Intervention Supporting Serious Illness Conversations in Medicaid-Funded Assisted Living

**DOI:** 10.1093/geroni/igaf122.1719

**Published:** 2025-12-31

**Authors:** Daniel David, Kimberly Hadson, Brianna Morgan, Kelseanne Breder, Neha Tungaturthy

**Affiliations:** New York University, New York, New York, United States; New York University, New York, New York, United States; New York University Langone Health, New York, New York, United States; New York University, New York, New York, United States; New York University, New York, New York, United States

## Abstract

Medicaid-funded assisted living programs (ALPs) provide essential support for under-resourced older adults who wish to age in place. However, these facilities lack structured support for serious illness management and advance care planning (ACP). As a result, residents experience significant health-related risks, including social isolation, unaddressed medical needs, avoidable hospitalizations, and premature nursing home placement. In this presentation we will present the findings from “Someone to Talk To,” a community health worker (CHW)-led intervention designed to facilitate serious illness conversations and introduce ACP among ALP residents. Through a five-year partnership with three NYC-based Medicaid-supported ALPs, qualitative research involving 75 low-income older adults and focus groups with staff and administrators major care gaps were identified. A resident-led Community Advisory Board (CAB) informed program development, emphasizing a strengths-based approach that prioritizes autonomy, trust-building, and meaningful engagement. “Someone to Talk To” pairs residents with CHWs under nurse supervision for three structured one-hour sessions addressing medical and social needs. The intervention incorporates the PREPARE for Your Care program, an evidence-based ACP tool designed for low-health-literacy populations. Key outcomes include: 1. Resident engagement in ACP discussions 2. Communication of ACP preferences with care teams 3. Development of a scalable, sustainable model for ACP integration in ALPs This pilot study assesses feasibility and “lessons learned”, laying the foundation for a broader implementation strategy to improve ACP engagement among low-income, high-risk older adults in ALPs.

